# Evaluation of Colombian silk fibroin hydrogels functionalized with recombinant LSECtin for intervertebral disc tissue engineering

**DOI:** 10.1371/journal.pone.0349634

**Published:** 2026-05-15

**Authors:** Lyda Cenobia Caballero-Méndez, Augusto Zuluaga-Vélez, Jhon Jairo Melchor-Moncada, Adrián Quintero-Martinez, Luc Mongeau, Sara Nejati, Juan Carlos Sepúlveda-Arias

**Affiliations:** 1 Grupo de Investigación Infección e Inmunidad, Facultad de Ciencias de la Salud, Universidad Tecnológica de Pereira, Pereira, Colombia; 2 Instituto de Química, Universidad Nacional Autónoma de México, Circuito Exterior, Ciudad Universitaria, CDMX, Coyoacán, Mexico; 3 Department of Mechanical Engineering, McGill University, Montreal, Canada; University of Eastern Piedmont: Universita degli Studi del Piemonte Orientale Amedeo Avogadro, ITALY

## Abstract

This study evaluated the potential of Colombian silk fibroin hydrogels functionalized with recombinant LSECtin (rLSECtin) for intervertebral disc tissue engineering. *In silico* analysis indicated that rLSECtin may interact with glycans similar to those found in human adipose-derived stem cells (ADSCs) through residues Asn105, Asp106, Glu93, Asn87, and Glu85, which coordinate calcium to form hydrogen bonds with GlcNAc hydroxyl groups. rLSECtin was successfully produced, purified, and incorporated into silk fibroin hydrogels. Thermal analysis showed that the hydrogels were resistant to sterilization, and rheological studies demonstrated predominantly elastic, gel-like behavior. The secondary structure of the hydrogels was largely determined by α-helical content, which is related to silk I with β-sheets also contributing as a result of sonication-induced structuring. Metabolic activity assays indicated that rLSECtin was associated with increased ADSC metabolic activity under two-dimensional conditions, whereas hydrogels containing rLSECtin maintained cell viability, as confirmed by cell viability assays. Differentiated constructs exhibited glycosaminoglycan-rich extracellular matrix and chondrocyte-like morphology, whereas rLSECtin-functionalized hydrogels showed increased transcription of *SOX9*, *ACAN*, and *COL2A1*, along with elevated *COL10A1* of uncertain significance for nucleus pulposus stability. Overall, Colombian silk fibroin hydrogels provide a mechanically stable and cytocompatible platform relevant for nucleus pulposus tissue engineering, in which rLSECtin appears to act primarily as a biochemical modulator rather than a structural modifier. These findings support a proof of concept for lectin-functionalized biomaterials in intervertebral disc regeneration. However, further studies, including mechanistic validation of glycan–lectin interactions, protein-level confirmation, quantitative extracellular matrix analysis, and evaluation of signaling pathways, are required to establish functional and translational relevance.

## Introduction

The intervertebral disc is a fibrocartilaginous structure that separates the vertebrae of the spine and plays a crucial role in supporting a complex combination of mechanical compressive and shear stresses during movement. This tissue comprises the nucleus pulposus (NP), the annulus fibrosus, and cartilaginous plates that anchor the discs to adjacent vertebrae [1]. Specifically, the nucleus pulposus is the soft, gelatinous central portion of the intervertebral disc that moves with changes in posture and is composed of collagen fibers, proteoglycans, glycoproteins, and chondrocyte-like nucleus pulposus cells (NPCs) [2].

Intervertebral disc degeneration (IVD) is a pathological process that leads to structural and functional alterations in tissues, which can result in significant disability and chronic pain [3]. According to global estimates, approximately 266 million people suffer from this condition, and it has been observed that 97% of lumbar discs undergo degeneration in individuals over 50 years of age [4,5]. IVD is characterized by a series of pathological alterations that affect the homeostasis of the intervertebral disc. These alterations include changes in gene expression and cellular function of NPCs as well as alterations in the composition and structure of the extracellular matrix. These changes can reduce the intervertebral disc's ability to absorb and distribute mechanical loads, compromising its structural and functional integrity [6].

To avoid total disc collapse in the IVD, hydrogels have been injected into the annulus fibrosus to replace the nucleus pulposus and preserve the disc. These implants swell in the presence of fluids *in vivo*, thereby radially loading the annulus fibrosus and separating the vertebrae [7]. The use of human adipose-derived stem cells (ADSCs), in this context, constitutes a promising option [8], because these cells have the ability to stably express genes after differentiation into the intervertebral disc and can exhibit mechanical characteristics similar to those of the native tissue.

In Latin American countries, the availability of such a therapeutic strategy remains limited [9,10]. To address this deficiency, it is essential to conduct research and develop products using local raw materials to enhance industrialization, improve healthcare services, and generate value chains in the region.

In Colombia, commercial silk is obtained exclusively from the cocoons of the Colombian silkworm hybrid Pílamo 2 because of its adaptation to local environmental and climatic conditions [11]. Colombian silk fibroin is a fibrillar protein that has been used as a biomaterial in tissue engineering to manufacture films, membranes, nanoparticles, porous scaffolds, and hydrogels [12–15]. Hydrogels made from Colombian silk fibroin have been shown to exhibit adequate biocompatibility, favorable mechanical properties, controllable biodegradation rate, and structural adjustability [16,17]. These hydrogels have been cellularized with mesenchymal stem cells and have shown the capacity to promote their differentiation into chondrocyte-like cells and to express markers such as *COL1A1*, which are of interest for intervertebral disc regeneration. It is well known that the TGF-β signaling pathway promotes the differentiation of mesenchymal stem cells towards chondrocytes and NPCs [18]. Therefore, the application of Colombian silk fibroin hydrogels for intervertebral disc regeneration requires specific stimuli that promote cellular processes, such as differentiation into NPCs.

Previous studies have shown that C-type lectins regulate the regeneration and maintenance of nucleus pulposus cells [19]. Functionalizing biomaterials with these molecules may be beneficial for intervertebral disc regeneration. C-type lectins are Ca^2+^-dependent proteins that recognize and bind cell surface terminal sugars with high specificity [20]. They are all homologous in the sequences and secondary structures of their carbohydrate recognition domains (CRDs). These lectins are among the best-known sugar-binding proteins [21]. Owing to their modularity, CRDs can be isolated from the rest of the proteins, and their specificity can be modified relatively easily. These characteristics make them ideal for immobilization on scaffolds to be used in tissue engineering.

Specifically, LSECtin (liver and lymph node sinusoidal endothelial cell C-type lectin) is a C-type lectin that binds mannose, N-acetylglucosamine (GlcNAc) and fucose, but not galactose. This lectin is mainly expressed in liver and lymph nodes, although data from Bgee (bulk RNA-Seq, scRNA-Seq, Affymetrix, in situ hybridization, EST data), establish that it is expressed in over 92 different tissues (https://www.bgee.org/gene/ENSG00000182566). From the point of view of its biological function, it is active as a virus receptor, in T cell proliferation and in immune response (https://www.uniprot.org/uniprotkb/Q6UXB4/entry). Results from LSECtin glycan arrays (www.functionalglycomics.org) revealed their potential to bind carbohydrate residues that are highly expressed on the surface of ADSCs, such as fucosylated Hex_2_HexNAc_2_Fuc_1_+Man_3_GlcNAc_2_, Hex_2_HexNAc_2_Fuc_1_NeuAc_1_ + Man_3_GlcNAc_2_, Hex_2_HexNAc_2_Fuc_1_NeuAc_2_ + Man_3_GlcNAc_2_ [22], and GlcNAcMan structures expressed on the surface of nucleus pulposus cells [23]. Therefore, in the present study, we sought to use the CRD of this protein, capable of recognizing specific sugars on the surface of ADSCs, to functionalize Colombian silk fibroin hydrogels and enhance the process of chondrogenic differentiation in the nucleus pulposus. Colombian silk fibroin was obtained for the manufacture of hydrogels. Once functionalized with recombinant LSECtin (rLSECtin), they were characterized and their potential *in vitro* for intervertebral disc regeneration was also evaluated.

## Materials and methods

### *In silico* analysis

rLSECtin (CRD of human LSECtin, UniProt ID: Q6UXB4) used in our assays was uploaded to the Boltz-1 (AlphaFold3) online server (https://neurosnap.ai/service/Boltz-1%20(AlphaFold3)) [24] together with two Ca^2+^ ions to generate an AlphaFold-based rLSECtin model in complex with Ca^2+^. The structure for GlcNAc(b1-2)Man(a1-3)[GlcNAc(b1-2)Man(a1-6)]Man(b1-4)GlcNAc(b1-4)[Fuc(a1-6)]GlcNAc (GlyCosmos ID: G80858MF) was obtained from the GLYCAM-web carbohydrate builder (https://glycam.org/), by checking the structure against that of GlyCosmos Entry G27919IH from PDB ID:6VSL.

Active and passive residues at the protein interfaces were obtained from analyzing the binding sites of PDB structures 6PWT and 6PWR. The docking was performed in HADDOCK2.4 (https://rascar.science.uu.nl/haddock2.4/). The solution with the best score showed interactions with terminal GlcNAc through hydroxyls at 3 or 4, which are clearly established as canonical for C-type lectins. The predicted interactions between the glycan and the protein were analyzed using the Arpeggio webserver [25]. In addition, interacting residues were depicted using LigPlot+ [26] to highlight the main contributions.

After docking, the model was processed using CHARMM-GUI [27] to dissolve it in an ionic medium mimicking Human Chondrogenic Differentiation Medium (Lonza) conditions (pH 7.4, 135 mM NaCl, 4 mM KCl, 1.5 mM CaCl_2_). Protonation states of residues were assigned at pH 7.4 using PROPKA [28] via CHARMM-GUI. The system was energy-minimized for 5000 steps using the steepest-descent algorithm to remove steric clashes and stabilize atomic coordinates. Convergence was confirmed by monitoring the potential energy gradient (Fmax < 1000 kJ/mol ∙ nm). The system was then equilibrated for 125 ps (125,000 steps, dt = 1 fs) with position restraints applied to heavy atoms (force constants: 400 kJ/mol·nm² for backbone, 40 kJ/mol·nm² for side chains). Temperature was maintained at a constant value using the v-rescale thermostat (τ = 1.0 ps). A subsequent 125 ps equilibration under isotropic pressure coupling (Parrinello-Rahman barostat, τ = 5.0 ps, P = 1 bar) was performed to stabilize system density. Position restraints were gradually relaxed. Finally, the binding free energy was computed using gmx_MMPBSA, analyzing trajectories from the equilibrated system to evaluate protein-ligand interactions.

### Production and purification of rLSECtin protein

rLSECtin was cloned from the pPB-T7-NcoI-RBS-LSECtin-XhoI vector (abm, S1 Appendix), transformed into *E. coli* DH5α (Thermo Fisher) at 50 ng/µL, and colonies were selected on LB agar with kanamycin (50 µg/ml). After minipreps, plasmid DNA was introduced into E. coli LEMO21(DE3) (NEB) at 28 ng/µL with selection on LB with kanamycin and chloramphenicol. For induction, colonies were grown in LB broth, adjusted to OD 0.4, and treated with IPTG at 400 µM for different concentrations of L-rhamnose and incubation times to define appropriate conditions for transformation. The cells were centrifuged, washed with 10 mM Tris-HCl, and stored at −80°C. To obtain protein, pellets were resuspended in 10 mM Tris-HCl, sonicated (20 W, 2 min), centrifuged (10,000 × g, 15 min), and solubilized with 6 M Guanidinium Chloride and β-mercaptoethanol (0.01%). After centrifugation, the proteins were solubilized in Triton X-100 (1%) and dialyzed (6000–8000 MWCO) for 12 h.

The purification was performed using a 1 mL CNBr-activated Sepharose 4B stationary phase (Cytiva), coupled to BSA (0.2 µg/µL) that had been previously glycated with mannose (ManBSA) via Maillard reaction [29]. The sample was recirculated overnight and eluted isocratically (0.5 mL/min) with 1.25 M NaCl, 25 mM Tris-HCl, and 2.5 mM EDTA, with detection at 214 nm. LSECtin activity was assessed using a fluorescence-based mannose competition assay with SYPRO Red-labeled mannose–BSA, as described in previous reports [30].

### Obtaining silk fibroin solutions

Silkworm cocoons of the Colombian hybrid *Bombyx mori L.* Pílamo 2 were collected from the experimental farm El Pílamo, Universidad Tecnológica de Pereira. Silk fibroin solutions were prepared using the conventional method described by Rockwood *et al*., with some modifications [31]. The silk cocoons were cleaned and cut into small pieces. A mass of 10 g of the cocoons was weighed and degummed in a Na_2_CO_3_ 0.02M solution at boiling for 30 min. Subsequently, the obtained material was washed with ultrapure water (UPW) generated using a Millipore Direct-Q8 purification system at room temperature. Fibroin was oven-dried at 40°C for 8–10 hours. Silk fibroin was solubilized at 20% (w/w) in 9.3M LiBr aqueous solution at 60°C for 24 h. The obtained solution was dialyzed with ultrapure water for 72 hours using 6000–8000 MWCO cellulose dialysis tubes (Fisherbrand). The fibroin solution was then autoclaved for 15 min at 121°C and centrifuged twice at 10,000 rpm for 25 min to remove the undissolved fibroin residues and aggregates. The final solution obtained was aliquoted and stored at 4°C for one week to prevent protein aging. The final silk fibroin concentration was determined by comparing the mass of the solution to that of the dried silk after drying at 60°C for 12 h.

### Fabrication of silk fibroin hydrogels with rLSECtin

The silk fibroin solution was prepared at a final concentration of 4% v/v using supplemented ADSC-GM SingleQuots cell culture medium, with rLSECtin protein at a final concentration of 4 µg/mL, in 2 mL tubes. This solution was sonicated for 3 s at a power rate of 80% using a PULSE 150 Ultrasonic Homogenizer (Benchmark) and subsequently incubated at 37°C for 5 min.

### Thermogravimetric analysis (TGA)

The thermal properties were studied using simultaneous TGA-DSC analysis on an SDT-650 (TA Instruments). Samples of the freeze-dried hydrogels (~3 mg) were loaded onto an alumina tray and heated in a nitrogen atmosphere from 25° to 500°C at a heating rate of 5 °C/min.

### Infrared spectroscopic analysis (FT-IR)

Infrared analysis was performed using Fourier transform infrared spectroscopy (FTIR) in the attenuated total reflectance (ATR) mode on a Cary 630 FTIR Spectrometer (Kaplan Scientific). Samples were analyzed under dry conditions, and spectra were acquired with 16 scans at a resolution of 4 cm^-1^ in the spectral region of 4000−400 cm^-1^ at room temperature. The secondary structure was quantified following the method proposed by Belton, with some modifications [32]. The spectra were loaded into Origin, and amide I bands between 1730 and 1600 cm^−1^ were analyzed. Using the FFT filter method, the band was adjusted to four Gaussian curves to calculate the percentage of contributing areas. These areas were assigned to the following secondary structures: α-helix (1618 cm^−1^), β-sheet (1646 cm^−1^), turns (1665 cm^−1^), and random coils (1696 cm^−1^).

### Swelling and degradation behavior of silk fibroin-based hydrogels

The swelling and degradation behavior of silk fibroin hydrogels, with and without rLSECtin, was evaluated following the procedure established by Bo et al. [33] with slight modifications. The dried sample was first weighed (W_0_), immersed in distilled water, and then removed at set time points. After the elimination of excess water, the wet weight (W_t_) was determined. The sample was dried again at 80 °C for 24 h in an oven and reweighed (W_2_). The swelling degree (S_W1_) was calculated as follows:


$$\begin{array}{c} S_{W\text{1}}=\frac{W_{t}-W_{\text{2}}}{W_{\text{0}}}\times \text{1}\text{0}\text{0}\% \end{array}$$

The degradation behavior of hydrogel samples was investigated by measuring cumulative protein release. Each sample was soaked in PBS solution at pH 7.4 and 37 °C. Supernatants were collected on days 1, 2, 3, 5, 7, and 8. For each collection, 200 µl of the supernatant was sampled. The cumulative amount of released protein in the collected fractions was quantified over time using the BCA method, and the optical density of the samples was measured at 562 nm using a Multiskan FC plate reader (Thermo Scientific, USA).

### Rheological and mechanical measurements

Mechanical characterization of the hydrogels was performed using a torsional rheometer (HDR-2, TA Instruments) with parallel plates (top-plate diameter of 20 mm) at room temperature. Cylinders of silk fibroin hydrogels and silk fibroin hydrogels with rLSECtin at a height of 5 mm and a diameter of 1.5 cm were used. To evaluate the dynamic viscoelastic properties of the hydrogels, frequency sweep measurements were performed in the range of 0.01–10 Hz, at 0.1% strain and 25°C. The storage (G’) and loss (G”) moduli were measured as a function of frequency to characterize the hydrogel's damping properties. Amplitude sweep tests were performed from 1% to 100% strain at 1 Hz to determine the linear viscoelastic region (LVER) and the yield point of the hydrogels. The crossover point G’/G” was evaluated from amplitude sweeps to estimate the brittleness of the hydrogels. The raw data for frequency and amplitude are presented in the Supplementary material (S5 Appendix).

### ADSC culture

Adipose-Derived Stem Cells (ADSCs) were cultured in ADSC-GM SingleQuots (LONZA) culture medium supplemented with gentamicin sulfate, Amphotericin-B, L-glutamine, and fetal bovine serum (FBS). Cells were incubated at 37 °C and 5% CO_2_ in culture media every 2 days.

### Cell viability assessment (Live & Dead Cell Staining)

Cell viability was evaluated using the Viability/Cytotoxicity Assay Kit for Animal Live & Dead Cells (Biotium, USA). Four treatments were tested: 1) ADSCs seeded directly into polystyrene boxes (control); 2) ADSCs cultured with rLSECtin 4 µg/mL; 3) ADSCs cultured on silk fibroin hydrogels; and 4) ADSCs cultured on silk fibroin hydrogels modified with rLSECtin 4 µg/mL. For all treatments, ADSCs were seeded onto 24-well plates at a density of 1  ×  10^4^ per well. After 24, 48, and 72 hours of incubation, the medium was replaced with 300 µL of calcein-AM/EthD-III, followed by 30 min incubation at 37 °C in the dark. After staining, samples were washed three times with PBS. Fluorescence images were acquired using a fluorescence microscope (Olympus, Japan). Quantitative analysis of cell viability was performed using ImageJ by calculating the ratio of live (green) to total cells.

### Measurement of Cell Metabolic Activity

Colombian silk fibroin-based hydrogels were used to assess potential effects on ADSC metabolic activity using the Alamar Blue assay [34]. The hydrogels were conditioned for 24 h prior to seeding stem cells, which were supplemented with ADSC-GM medium. S4-passage ADSCs were used in this study. Cells were seeded in 96-well plates at a density of 1500 cells/well under the same conditions previously used to test cell viability.

All the samples were incubated for 24h, 48h and 72h. The culture medium was aspirated from each well and replaced with 200 µL of 10% Alamar Blue reagent. The plates were incubated for an additional 4h at 37°C, 100 µL of each sample was added to the opaque polystyrene plates. The fluorescence of each well was measured at an excitation wavelength of 570 nm and emission wavelength of 585 nm using a Cary Eclipse fluorescence spectrophotometer (Varian).

### Chondrogenic differentiation of ADSCs

ADSC cells were expanded in feeder medium to a cell density of 1 x 10^7^ cells/mL, seeded at 5.0 x 10^5^ cells/mL in 15 mL tubes. Three types of samples were evaluated in triplicate: 1) hADSCs cell pellets; 2) cell pellets on silk fibroin hydrogels; and 3) cell pellets on silk fibroin hydrogels with rLSECtin 4 µg/mL. The cells were washed twice in incomplete chondrogenic medium, centrifuged at 150 g for 5 min at room temperature, and pellets were resuspended in Human Chondrogenic Differentiation Medium supplemented with TGF-β3 (Lonza). The cell culture medium was changed every two days for 21 days under standard conditions [35]. The resulting cultures were stained with Alcian Blue. The cells were frozen in liquid nitrogen, and total RNA was extracted using TRIzol Reagent (Life Technologies, New York, NY, USA). Gene expression levels of *ACAN* (Hs00153936), *COL2A1* (Hs0024051), *COL10A1* (Hs00166657), *SOX9* (Hs01001343), *COL1A2* (Hs00164099), and the housekeeping control gene *GAPDH* (Hs02758991) were evaluated using TaqMan Gene Expression assays (Thermo Fisher Scientific, USA). Total RNA was diluted in water, quantified using a NanoDrop2000, and stored at −80 °C. RT-qPCR was performed using the One-Step RT-PCR Kit (Applied Biosystems, USA) applying the 2^−ΔΔCt^ method. All results were normalized to gene expression in ADSCs cultured without exposure to differentiation medium (undifferentiated control).

### Statistical analysis

Results are presented as mean ± standard deviation (SD). Experiments were performed with at least three independent biological replicates, unless otherwise specified. Statistical analyses were performed using GraphPad Prism (version 10.4). For metabolic activity assays, two-way ANOVA followed by Tukey’s post hoc test was applied. For gene expression analysis, nonparametric Kruskal–Wallis tests, followed by Dunn’s multiple comparisons test, were used. A value of p < 0.05 was considered statistically significant.

### Ethics statement

Human adipose-derived stem cells (ADSCs) used in this study were obtained from a commercial supplier (Lonza). According to institutional and international guidelines, the use of commercially available human cell lines does not require ethical approval; however, this study was approved as “research without risk” by the Ethics Committee from Universidad Tecnológica de Pereira (Approval number 04–180520, May 20, 2020).

## Results and discussion

### *In silico* analysis

Although it has been reported that the isolation of the CRD does not affect the interaction between lectin and ligand [36], we decided to follow a computer-assisted approach to elucidate the binding mode of the rLSECtin protein to GlcNAc(b1-2)Man(a1-3)[GlcNAc(b1-2)Man(a1-6)]Man(b1-4)GlcNAc(b1-4)[Fuc(a1-6)]GlcNAc, as a glycan structurally related to those present on ADSCs [37].

The three-dimensional protein structure was generated using an AlphaFold3 server and was folded in the presence of calcium. The initial pose was determined by considering the known binding site for C-type lectins [38].

**Fig 1:**
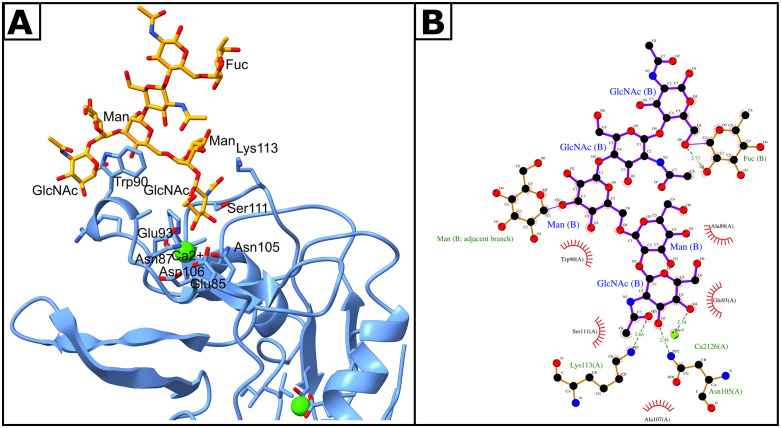
*In silico* analysis. **(A)**. Structural representation of the optimal docking result from HADDOCK demonstrating the binding interaction between the *carbohydrate recognition domain* (*CRD*) of LSECtin (rLSECtin) and the glycan ligand GlcNAc(b1-2)Man(a1-3)GlcNAc(b1-2)Man(a1-6)Man(b1-4)GlcNAc(b1-4)Fuc(a1-6)GlcNAc. The CRD is depicted in blue, while the glycan ligand is highlighted in orange, with key amino acid residues involved in the binding interactions annotated. ***(B)***. LigPlot+ depiction of the same interaction, illustrating interaction networks, particularly emphasizing the role of the Ca^2*+*^ ion located within the canonical binding site, which is essential for ligand binding stability. Notably, secondary interactions, including those by Trp90 with a mannose residue, enhance the structural integrity and specificity of the glycan-lectin interaction.

Subsequent poses were selected based on docking program scores. In addition, we limited the candidates to those in which the protein and glycan hydroxyls at positions 3 and 4 of the sugars interacted through a calcium ion, as this is the canonical binding mode of C-type lectins [39]. The main interaction occurred with a terminal GlcNAc(b1-2)Man, which is in agreement with the results reported by Bertuzzi et al. [40]. The results showed that residues Asn105, Asp106, Glu93, Asn87, and Glu85 were responsible for coordinating the calcium through which hydrogen bonds are formed with the GlcNAc hydroxyl (Fig 1). The glycan is stabilized through the presence of secondary interactions between Lys113 and the Man residue contiguous to the terminal GlcNAc, which establishes a hydrogen bond with the Ca^2+^, and π-π* interactions between Trp90 and the most distal core Man.

Additionally, rLSECtin-glycan interactions were evaluated by computing the binding free energy, and analyzing the trajectories after energy minimization and position-restrained equilibration. At equilibrium, the glycan barely moved from the binding site, forming a stable system. These findings indicate that rLSECtin may interact favorably with glycans representative of those found on ADSCs (S2 Appendix). The simulation revealed a net favorable interaction energy of −8.97 kcal/mol. Despite the polar solvation penalty, the net binding is stabilized by strong van der Waals interactions, typical of buried binding pockets. This concurs with findings regarding the integrity of C-type lectin binding site [41]. However, these findings are based solely on computational predictions and therefore do not constitute direct biological evidence.

### rLSECtin production and separation

Genetic transformation of the bacterium *LEMO21* with the plasmid pPB-T7-NcoI-RBS-LSECtin-XhoI was performed to express rLSECtin. Optimization of inducer concentration and total induction time was conducted to maximize rLSECtin production under controlled laboratory conditions. The optimal conditions for rLSECtin overexpression in the present system were a 100 µM L-rhamnose concentration and an induction time of 8 h (S3 Appendix). Previous studies [42] on the production of recombinant lectins have shown that optimizing expression parameters is essential due to the high variability across expression systems and their effects on protein solubility, aggregation, and functionality. It should be noted that, in addition to the strategy employed in this study, more advanced optimization methodologies, such as Plackett-Burman or Taguchi experimental designs, can be implemented to systematically identify the most efficient parameters for specific protein production in rLSECtin bacterial culture conditions [43].

**Fig 2:**
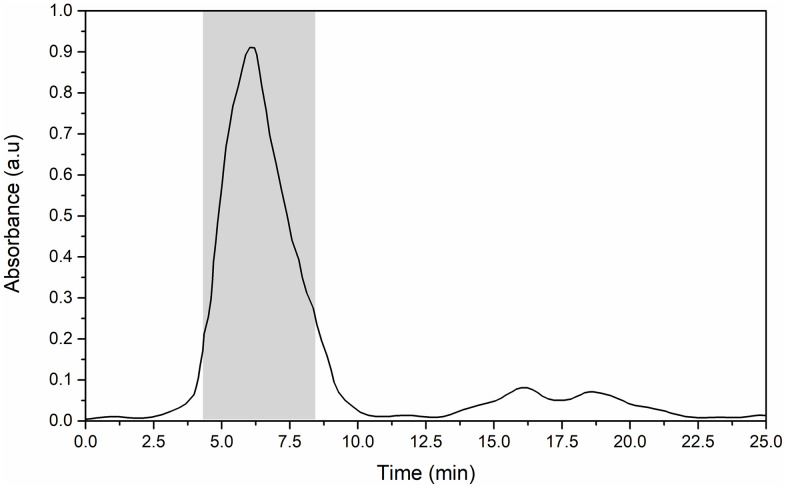
Purification of rLSECtin. Elution profile of rLSECtin purified by affinity chromatography on ManBSA functionalized Sepharose. The gray shaded area corresponds to the purified rLSECtin fractions. rLSECtin was eluted with 1.25 M NaCl, 25 mM Tris-HCl, and 2.5 mM EDTA at a flow of 0.5 mL/min.

Fig 2 shows the chromatographic elution profile of rLSECtin purified by affinity chromatography to ManBSA. The elution peak observed between 4.0 and 8.5 minutes (gray-shaded region) corresponds to the purified rLSECtin fractions. The sharpness of the major peak indicates efficient protein recovery, whereas minor broadening near 8.0 minutes suggests trace nonspecific interactions. Notably, LSECtin’s affinity for mannose parallels that of other C-type lectins such as DC-SIGN2, which also bind high-mannose glycans but differ in tissue distribution and immune functions [21]. Importantly, the use of mannose-functionalized matrices not only enabled purification but also provided indirect evidence of the recombinant CRD domain's preserved carbohydrate-binding activity. This was additionally confirmed by solid-phase competition assays that tested the binding to glycated BSA via mannosyl residues (S4 Appendix).

### Hydrogel characterization

**Fig 3:**
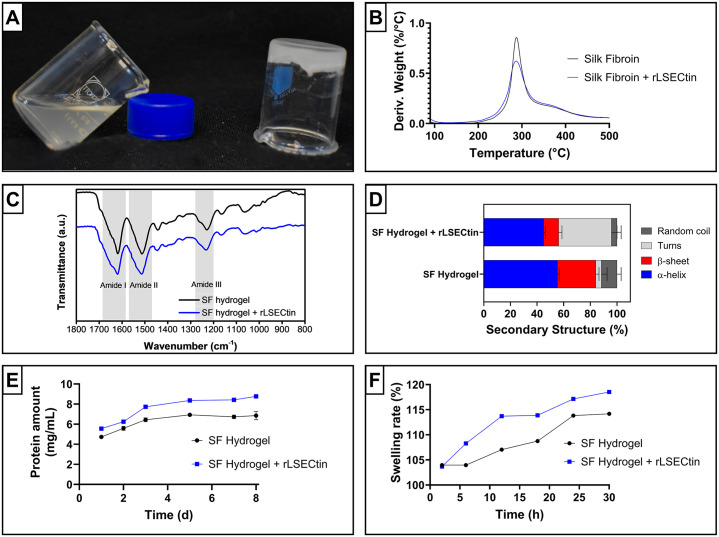
Physicochemical characterization of silk fibroin-based hydrogels. **(A)**. Photograph showing silk fibroin in solution and silk fibroin-based hydrogel. **(B)**. Thermal analysis, **(C)**. ATR-FTIR spectra, and **(D)**. deconvolution analysis of the amide I band of silk fibroin hydrogel and rLSECtin-functionalized silk fibroin hydrogel.

Sterile solutions of silk fibroin and rLSECtin were used to fabricate hydrogels (Fig 3A). Thermal analysis showed that Colombian silk fibroin was resistant to the sterilization temperature (121 °C) and that mixing with the recombinant CRDs did not affect the maximum local degradation rate, which was approximately 287 °C (Fig 3B). This is in agreement with previous observations on the relationship between temperatures of 280–290 °C and the degradation of polypeptide chains via cleavage of peptide bonds, amino acid residues, and side chains [44].

The ATR-FTIR analyses (Fig 3C) showed that the silk fibroin hydrogels and modified silk fibroin hydrogels possess the band features amide I (1590−1690 cm^-1^), amide II (1480−1570 cm^-1^), and amide III (1200−1275 cm^-1^) related to the elongation vibrations of C = O, C-H, and C-N bonds, bending of N-H bonds, and vibrations of polypeptide chain side chains, respectively [45]. As demonstrated by other authors, the shift of the amide I band towards 1625 cm^-1^ is attributed to the formation of the β-sheet structure of silk fibroin after gelation by the sol-gel process [46].

To evaluate the secondary structure content of the proteins in the hydrogels, deconvolution of the amide I peak (Fig 3D) was performed by decomposing the signal into four components: α-helix (1618 cm − 1), β-sheet (1646 cm − 1), turns (1665 cm − 1), and random coils (1696 cm − 1), as reported by Belton [32]. The results of this analysis show that the secondary structure of the hydrogels is largely determined by an alpha-helix structure (45-55%), which is related to silk I, a metastable form that is soluble and poorly crystalline [47]. These structural motifs combine to form a circular pattern in the amorphous orthorhombic system, resulting in silk I, a thermodynamically unstable phase [48].

In silk fibroin hydrogels, β-sheets are the second most abundant secondary structure, and their formation is attributed to sonication. This process can induce the formation of nano-crystals composed of intermolecular β-sheets organized into semi-crystalline arrays that significantly contribute to the structural stability of the material [49,50]. The incorporation of the CRD domain into the hydrogels significantly affected the turns content within the material structure. Through solid-state NMR studies, some authors have related the loop and turn content in silk fibroin-based materials to the adoption of a lamellar structure [51]. Similarly, the Alphafold3-predicted model of the recombinant CRD shows the presence of disordered structures and loops that can impact the flexibility and assembly of β-sheets within the material. Some authors have reported that certain molecules, such as curcumin and β-cyclodextrin, can also modulate the self-assembly of nanofibrils during the formation of hierarchical structures. This effect is mainly due to the fact that these molecules sequester monomeric units within their hydrophobic cavities, which prevents further aggregation and regulates nanofibril growth [52].

Since hydration capacity and controlled material stability are critical parameters for nucleus pulposus replacement strategies, swelling and degradation assays were performed. Swelling assays reached equilibrium after ~24 h, with values of 115–120% and no statistically significant differences between groups (Fig 3F). This indicates that rLSECtin does not substantially alter bulk network properties. Such moderate swelling may be advantageous in confined environments such as the intervertebral disc, where excessive expansion could be detrimental [53]. Degradation analysis revealed protein release between 5 and 8 mg/mL over 3 days (Fig 3E), suggesting controlled matrix stability [54].

**Fig 4:**
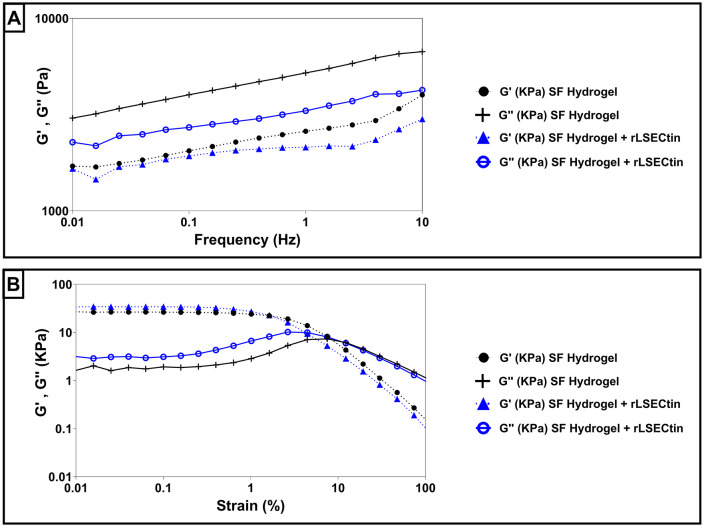
Rheological analyses of silk fibroin hydrogel and rLSECtin-functionalized silk fibroin hydrogel. **(A)**. Frequency sweep dynamic rheological data. **(B)**. Amplitude sweep tests.

Rheological analyses of silk fibroin hydrogels, both in the presence and absence of rLSECtin, revealed that the storage modulus (G′) remained higher than the loss modulus (G″) across the entire frequency range tested (Fig 4A). This relationship (G′ > G″) indicates predominantly elastic, gel-like behavior and reflects strong molecular interactions within the three-dimensional hydrogel network that resist flow or molecular slippage.

In amplitude sweep tests, the linear viscoelastic region (LVER) was clearly identified for all samples, and the yield point was determined from the intersection of G′ and G″ (Fig 4B). In both types of hydrogels (without and with rLSECtin), the crossover occurred at a relatively low strain—close to 10%—suggesting that these materials exhibit a fragile or brittle network. Once this critical strain limit is surpassed, the hydrogel network structure breaks down, and both G′ and G″ become highly sensitive to further increases in strain [55]. Overall, these findings demonstrate that silk fibroin hydrogels, irrespective of rLSECtin incorporation, exhibit predominantly elastic behavior under small deformations, with a well-defined yield point marking the onset of structural breakdown. This behavior can be attributed to the dense cross-linked network of silk fibroin, which confers high elasticity up to a relatively low strain threshold (Fig 4). Notably, the incorporation of rLSECtin into the silk fibroin matrix did not shift the yield point, implying that the fundamental cross-linked fibroin network dominates the mechanical response.

### Cell Viability and Metabolic Activity in Silk Fibroin Hydrogels Functionalized with rLSECtin

**Fig 5:**
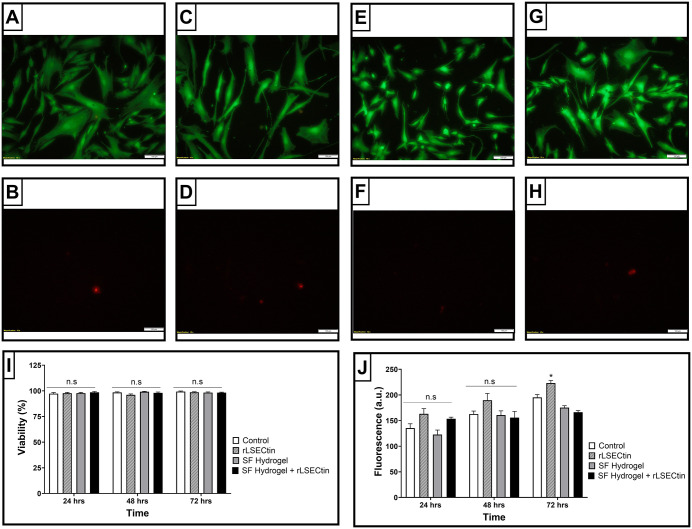
Viability and metabolic activity of ADSCs on silk fibroin-based hydrogels measured at different time points. ***(A-H)***. Live & Dead Cell Staining assay of ADSCs treated for 3 days. ADSCs were stained with Calcein AM (live cells, green fluorescence) and EthD-III (dead cells, red fluorescence). ***(A-B)***. Control group; ***(C-D)***. ADSCs treated with rLSECtin; ***(E-F)***. Cells cultured on silk fibroin hydrogels; ***(G-H)***. Cells cultured on silk fibroin hydrogels modified with rLSECtin. ***(I)***. Statistical analysis of the cell viability of ADSCs after different treatments. ***(J)***. Statistical analysis of the cell metabolic activity of ADSCs after different treatments. Error bars in (I-J) indicate standard deviations of triplicate measurements. Data in (I-J) were analyzed by two-way ANOVA followed by a post hoc Tukey test (n.s., not significant); (*, p < 0.05).

Live/Dead assays (Fig 5A–I) showed high viability across all groups. A predominance of viable cells was observed at all time points (24–72 h), with no increase in cell death compared to controls.

Quantitative analysis confirmed no significant differences between groups, indicating that both native and functionalized silk fibroin hydrogels are cytocompatible. These findings indicate that degradation products do not induce cytotoxic effects, in contrast to previous reports [56]. The low cytotoxicity may be attributed to the use of the isolated CRD domain, which avoids regions of the structure associated with additional intracellular effects [57].

Alamar Blue results showed no significant differences in metabolic activity (Fig 5J). This suggests that rLSECtin does not enhance proliferation under 3D conditions. Importantly, metabolic activity in 3D systems does not directly correlate with proliferation, as cells may adopt a quiescent phenotype [58]. This is consistent with nucleus pulposus biology [59]. The slight reduction in signal observed in functionalized silk fibroin hydrogels may therefore reflect metabolic quiescence rather than cytotoxicity.

ADSCs cultured in 2D with rLSECtin showed increased metabolic activity, consistent with mTORC1 activation [60]. However, this effect was not reproduced in 3D, likely due to differences in protein accessibility and spatial distribution. These findings highlight the importance of biomaterial architecture in regulating protein bioavailability and cell responses.

The results obtained with silk fibroin hydrogels with rLSECtin contrast with those of other studies, such as those performed with macroporous hydrogels [61]. In these systems, the lectin-mediated immobilization of human cells, such as YFP-LecB, has been shown to enable effective cell proliferation within the 3D matrix while maintaining cell morphology and viability. Unlike silk fibroin hydrogels, this system offers adjustable pores in the micrometer range, which facilitates the creation of suitable niches for cell culture. This contrast may be associated with structural differences between hydrogel systems, including pore architecture and protein distribution within the matrix. In the present system, sonication-induced gelation may lead to a heterogeneous distribution of rLSECtin, potentially limiting its accessibility to interact with cell-surface glycans. Additionally, sonication may influence protein conformation or oligomerization, which could affect lectin functionality [62,63]. However, these factors were not directly evaluated in this study.

Future studies should evaluate focal adhesion markers (vinculin, paxillin), integrins (CD44, CD29), and signaling pathways (ERK, AKT, SMAD) to establish mechanistic links [62]. Additionally, evaluation under inflammatory conditions (IL-1β, TNF-α) would improve translational relevance by better reflecting the inflammatory microenvironment of the intervertebral disc [63].

### Differentiation of ADSCs towards chondrocyte-like NP cells on silk fibroin-based hydrogels

**Fig 6:**
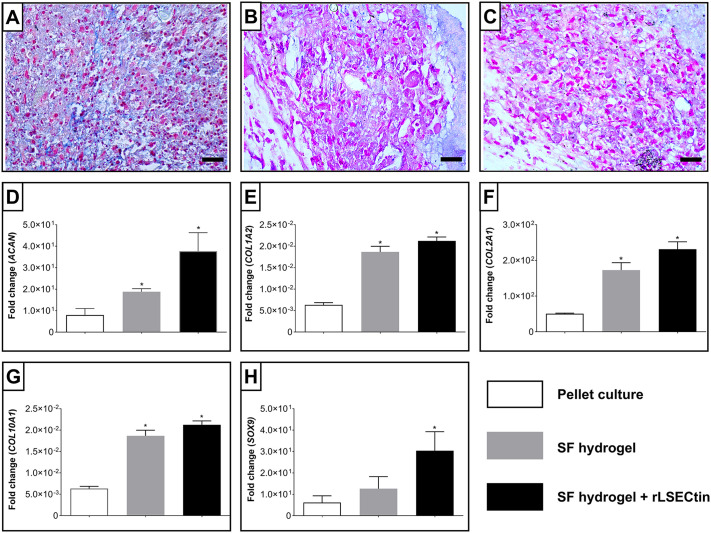
Histological and molecular evaluation of ADSC differentiation. Alcian blue staining of: **(A)**. pellet culture, **(B)**. pellet culture on silk fibroin hydrogel, and **(C)**. pellet culture on rLSECtin-functionalized silk fibroin hydrogel. Scale bar: 500µm. Chondrogenic gene expression profiles of (D). ACAN, (E). COL1A2, (F). COL2A1, (H). COL10A1, and (I). SOX9. Error bars indicate standard deviations of triplicate measurements. Kruskal–Wallis test followed by a post hoc Dunn test. (n.s., not significant); (*, p < 0.05).

In Fig 6A-C intense bluish staining of the extracellular matrix was observed in all groups, indicating the abundant presence of glycosaminoglycans, which are characteristic components of the nucleus pulposus [64]. The presence of sulfated glycosaminoglycans around the cells is consistent with a chondrogenic-like differentiation process towards a nucleus pulposus phenotype. In addition, scattered cells with rounded morphology were grouped into small clusters, as for chondrocyte-like cells [65]. Some authors have also reported that ADSC differentiation is adequate after three weeks of culture, with significant production of GAGs, thereby increasing the water content and height of the nucleus pulposus, which may slow the progression of IVD [66]. In fact, some authors have reported that, in silk fibroin-based biomaterials, the presence of GAG increases over time and correlates with an increase in the compressive elastic modulus [67]. Additionally, as reported in the literature, some C-type lectins, such as langerin, have the ability to recognize glycosaminoglycans [68], which could impact the production or deposition process. However, no marked qualitative differences in GAG staining were observed between the different hydrogels. This may be related to the intrinsic stability of silk fibroin as a matrix and the possible saturation of available binding sites within the material, as reported for similar systems [69]. It should be noted that Alcian Blue staining provides qualitative rather than quantitative information, and therefore does not allow definitive comparison of extracellular matrix production between groups. Additionally, as reported by other authors [67], it could be considered relevant to evaluate their accumulation over time in hydrogels to reveal differences in the regeneration process and the efficiency of the construct.

The expression of genes characteristic of chondrogenesis was evaluated after 21 days of culture in a differentiation medium containing TGF-β (Fig 6D-I). Data were analyzed using the nonparametric Kruskal-Wallis test, followed by Dunn's multiple comparison test. All results were normalized to gene expression in ADSCs not exposed to the differentiation medium (undifferentiated control). ADSCs cultured within Colombian silk fibroin hydrogels exhibited transcriptional profiles consistent with chondrogenic differentiation, including the expression of *ACAN*, *COL1A2*, and *COL2A1,* in agreement with previous reports. The inclusion of rLSECtin was associated with increased expression of *SOX9*, a key transcription factor in cartilage development and homeostasis [70], as well as increased expression of *COL2A1* and *ACAN*, at the transcriptional level. Taken together, these findings suggest that cells within Colombian silk fibroin-based hydrogels may adopt a phenotype consistent with extracellular matrix synthesis associated with nucleus pulposus-like cells. These genes encode extracellular matrix components that are known to contribute to the mechanical resilience and diffusional properties of native nucleus pulposus tissue.

In contrast, we observed that *COL10A1* expression increased with the incorporation of rLSECtin, relative to the differentiated cell pellet. *COL10A1* is a marker of hypertrophic chondrocyte differentiation in articular cartilage [71]. However, recent transcriptomic studies have also reported its expression in both articular cartilage and nucleus pulposus tissues [72]. Therefore, the biological significance of *COL10A1* expression in this system remains unclear, particularly when compared to its expression in undifferentiated stem cells.

Furthermore, in the absence of protein-level validation or additional functional assays, it is not possible to confirm whether this finding reflects the establishment of a stable nucleus pulposus-like phenotype.

### Hydrogel-based systems in Intervertebral Disc tissue engineering

Hydrogels are bioactive biomaterials that modulate mesenchymal stem cell differentiation through biodegradability, stiffness, morphology, and degradation by-products [73]. In intervertebral disc regeneration, they serve as dynamic platforms for delivering drugs, RNA interference systems, exosomes, and proteins [74–76]. Silk fibroin-based hydrogels are used for nucleus pulposus repair, incorporating bioactive factors [77,78] and promoting extracellular matrix regeneration and immunomodulation [79,80]. Beyond regenerative applications, hydrogel-based systems have been used for cell separation and purification, emphasizing the need to better understand cell–matrix interactions and molecular transport in these environments [81–83]. However, key aspects such as cell migration and diffusion remain insufficiently characterized. In this context, the present study provides proof of concept for lectin-functionalized hydrogels while highlighting the need for further mechanistic and functional validation.

## Conclusions

Silk fibroin-based hydrogels derived from the Colombian hybrid Pílamo 2, functionalized with the recombinant protein rLSECtin, represent a promising proof-of-concept platform for intervertebral disc tissue engineering applications. The incorporation of rLSECtin preserved the physicochemical and mechanical properties of the hydrogels while maintaining cytocompatibility within the three-dimensional matrix.

At the transcriptional level, ADSCs cultured within these hydrogels exhibited gene expression profiles consistent with a chondrogenic-like phenotype, including upregulation of *SOX9*, *COL2A1,* and *ACAN*. These observations, together with glycosaminoglycan staining, suggest that the material may support early stages of differentiation towards nucleus pulposus-like cells. However, these findings are based on transcriptional and qualitative analyses and do not constitute direct evidence of functional extracellular matrix deposition or the acquisition of a stable phenotype.

In addition, *in silico* analysis further suggests a potential interaction between rLSECtin and glycans present on ADSC surfaces. However, this mechanism has not been experimentally validated and would require glycan competition assays or calcium-chelation experiments in the presence of ADSCs or the use of complex synthetic glycans. Taken together, these results indicate that rLSECtin primarily acts as a biochemical modulator of cell behavior rather than a structural modifier of the hydrogel system, supporting the feasibility of incorporating lectin-derived domains into biomaterial platforms. Nevertheless, key limitations include the lack of experimental validation of glycan–lectin interactions, protein-level validation, and intracellular signaling analysis.

Overall, within the broader context of bioactive hydrogel-based systems for tissue engineering, this work provides proof of concept for lectin-functionalized silk fibroin hydrogels in intervertebral disc regeneration. Further studies incorporating protein-level validation, quantitative extracellular matrix analysis, and mechanistic investigation are required to establish their biological and translational relevance.

## Supporting information

S1 Appendix Cloning vector pPB-T7-NcoI-RBS-rLSECtin-XhoI. The fragment encoding rLSECtin is shown in green.
(TIF)

S2 Appendix System equilibration monitoring. (A). Potential energy convergence during steepest descent minimization (5000 steps). The gradient F max < 1000 kJ/mol·nm confirmed stable atomic coordinates. (B). Temperature stability (303.15 K) during NVT equilibration (125 ps) with position restraints on heavy atoms (backbone: 400 kJ/mol·nm²; side chains: 40 kJ/mol·nm²), regulated by the v-rescale thermostat (τ = 1.0 ps). (C). Pressure equilibration (1 bar) under NPT conditions (Parrinello-Rahman barostat, τ = 5.0 ps) with gradual restraint relaxation. Panels A–C demonstrate successful equilibration.
(TIF)

S3 Appendix SDS-PAGE analysis of the induction conditions for rLSECtin production. M: Molecular weight marker. Lane 1: 10 hours at 0 μM, Lane 2: 8 hours at 2000 μM, Lane 3: 8 hours at 1000 μM, Lane 4: 8 hours at 500 μM, Lane 5: 8 hours at 100 μM, Lane 6: 8 hours at 0 μM, Lane 7: 6 hours at 2000 μM, Lane 8: 6 hours at 1000 μM, Lane 9: 6 hours at 500 μM. The concentrations refer to L-rhamnose. The red box identifies rLSECtin bands.
(TIF)

S4 Appendix Fluorescence-based solid-phase competition assay of LSECtin activity. Microplates were coated with rLSECtin, and increasing concentrations of mannose were evaluated as a competing ligand in the presence of SYPRO Red-labeled mannose-BSA (glycated) as a reporter. Fluorescence signals were measured using a Cytation 3 Cell Imaging Multi-Mode Reader.
(TIF)

S5 Appendix Raw data from rheology experiments (frequency and amplitude). (PDF)
