# Supplementary figures and images for: Evaluation of Colombian silk fibroin hydrogels functionalized with recombinant LSECtin for intervertebral disc tissue engineering
Source: PLoS One. 2026 May 15;21(5):e0349634. doi: 10.1371/journal.pone.0349634 (PMC13178859; doi:10.1371/journal.pone.0349634)

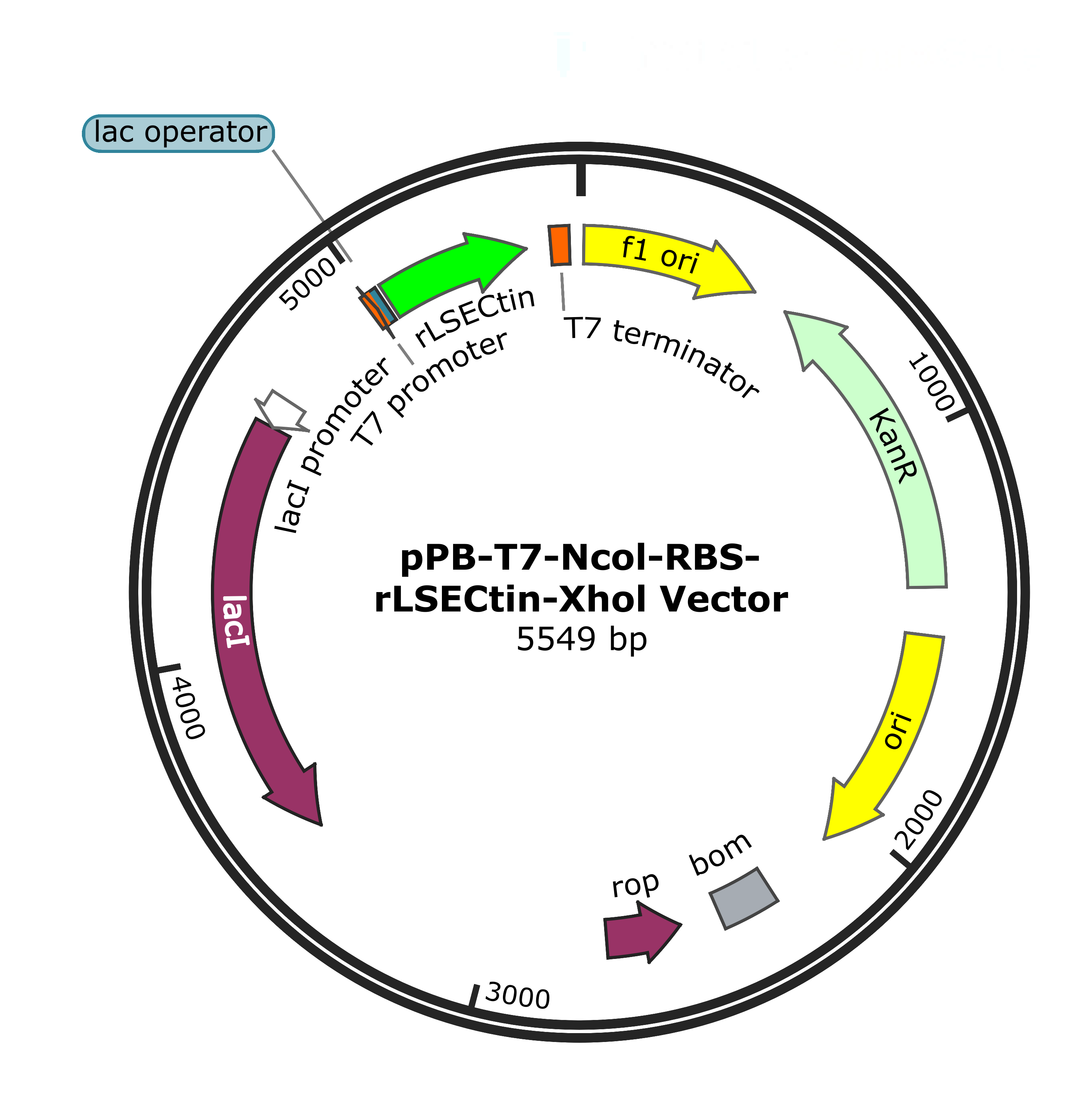

Supplement: S1 Appendix — The fragment encoding rLSECtin is shown in green. (TIF) [file pone.0349634.s001.tif]

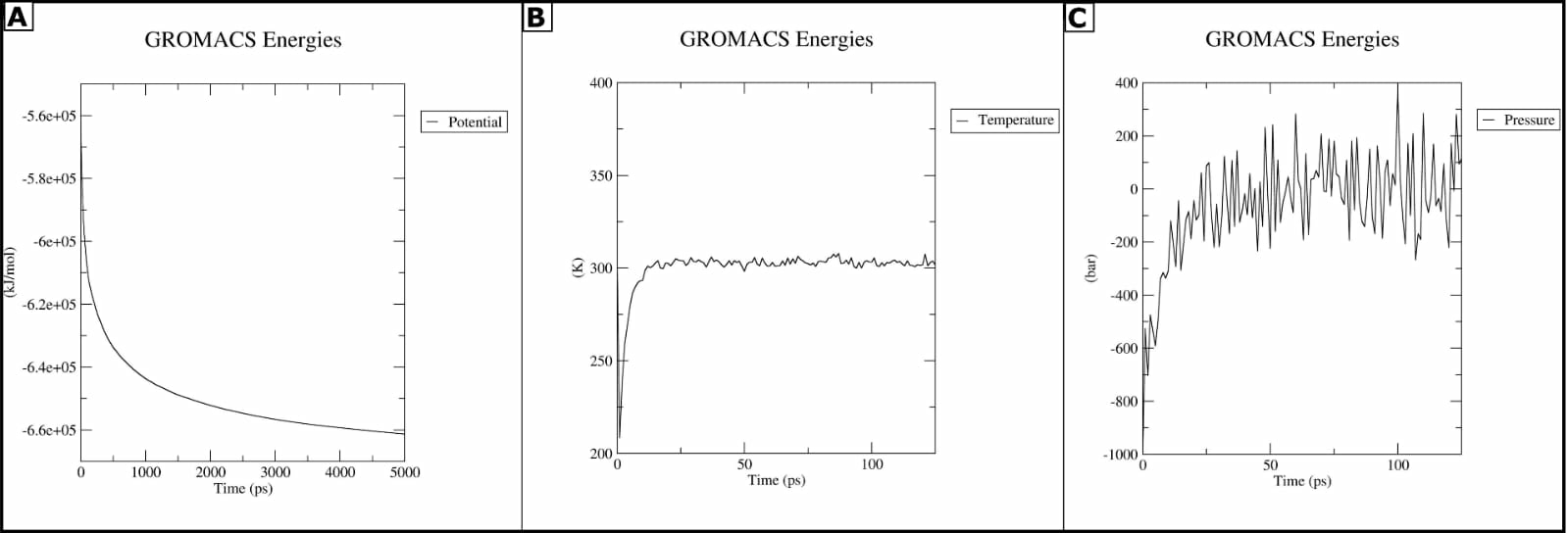

Supplement: S2 Appendix — (A). Potential energy convergence during steepest descent minimization (5000 steps). The gradient F max < 1000 kJ/mol·nm confirmed stable atomic coordinates. (B). Temperature stability (303.15 K) during NVT equilibration (125 ps) with position restraints on heavy atoms (backbone: 400 kJ/mol·nm²; side chains: 40 kJ/mol·nm²), regulated by the v-rescale thermostat (τ = 1.0 ps). (C). Pressure equilibration (1 bar) under NPT conditions (Parrinello-Rahman barostat, τ = 5.0 ps) with gradual restraint relaxation. Panels A–C demonstrate successful equilibration. (TIF) [file pone.0349634.s002.tif]

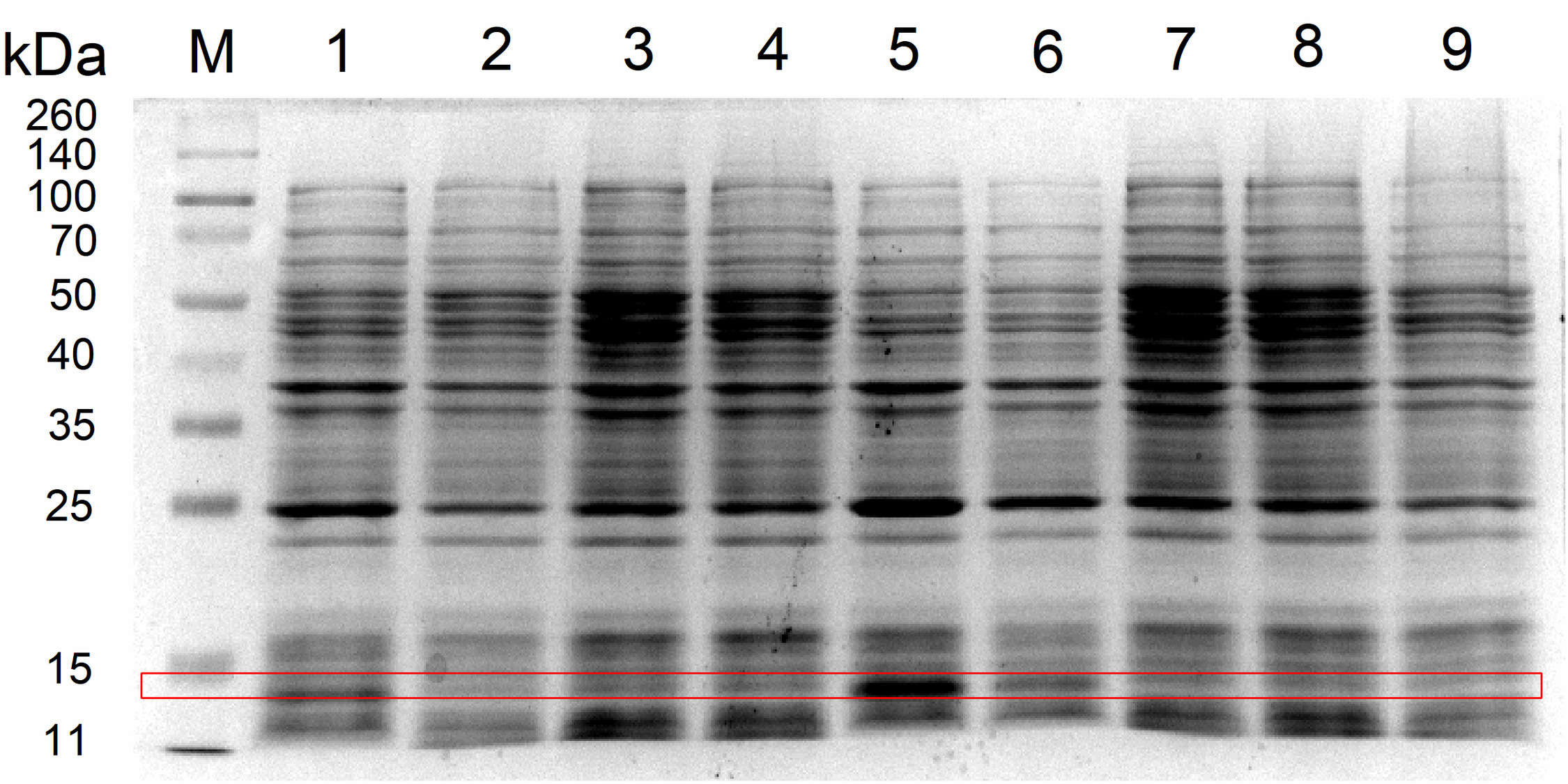

Supplement: S3 Appendix — M: Molecular weight marker. Lane 1: 10 hours at 0 μM, Lane 2: 8 hours at 2000 μM, Lane 3: 8 hours at 1000 μM, Lane 4: 8 hours at 500 μM, Lane 5: 8 hours at 100 μM, Lane 6: 8 hours at 0 μM, Lane 7: 6 hours at 2000 μM, Lane 8: 6 hours at 1000 μM, Lane 9: 6 hours at 500 μM. The concentrations refer to L-rhamnose. The red box identifies rLSECtin bands. (TIF) [file pone.0349634.s003.tif]

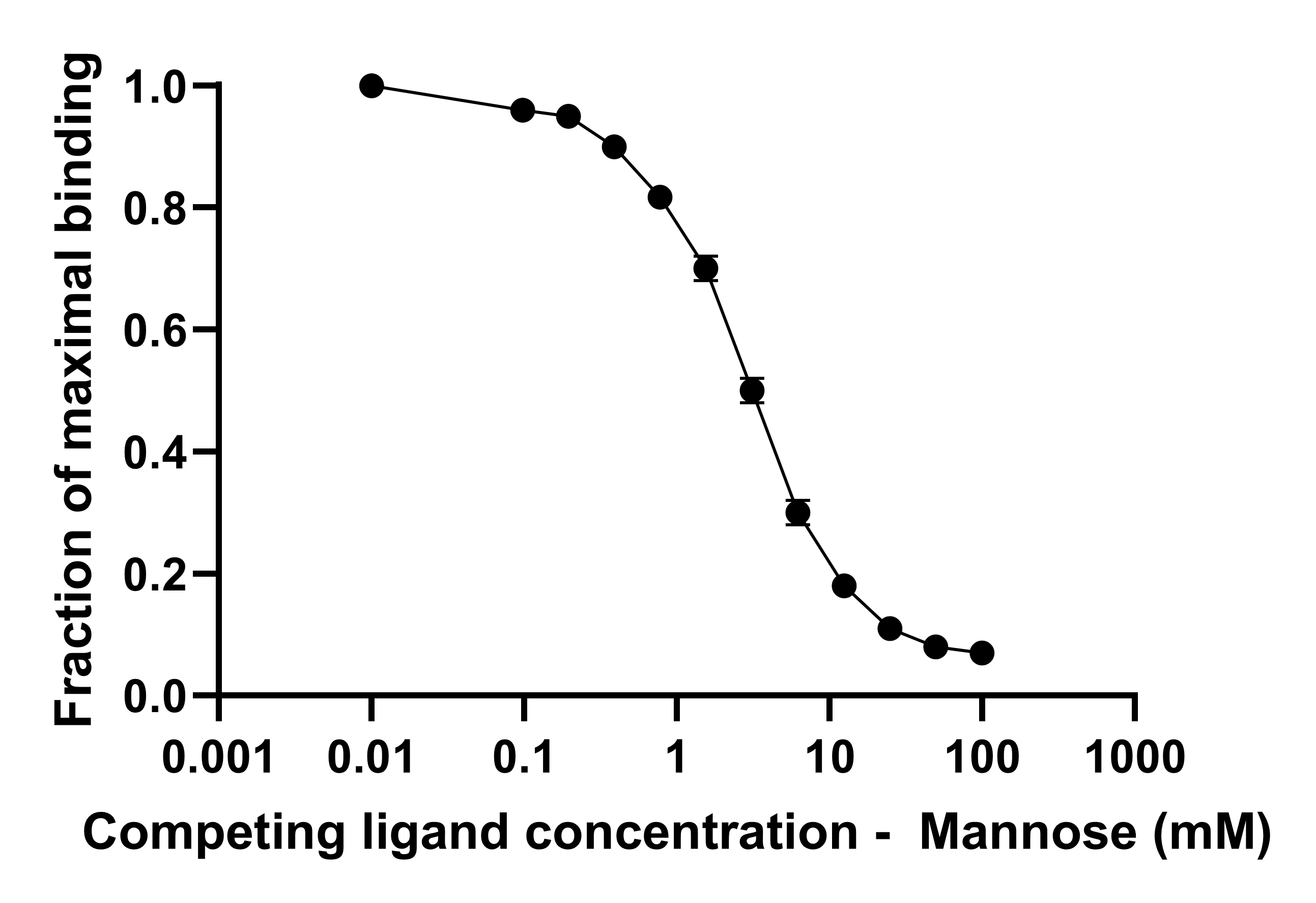

Supplement: S4 Appendix — Microplates were coated with rLSECtin, and increasing concentrations of mannose were evaluated as a competing ligand in the presence of SYPRO Red-labeled mannose-BSA (glycated) as a reporter. Fluorescence signals were measured using a Cytation 3 Cell Imaging Multi-Mode Reader. (TIF) [file pone.0349634.s004.tif]
