# Supplementary material for: Evaluation of Colombian silk fibroin hydrogels functionalized with recombinant LSECtin for intervertebral disc tissue engineering
Source: PLoS One. 2026 May 15;21(5):e0349634. doi: 10.1371/journal.pone.0349634 (PMC13178859; doi:10.1371/journal.pone.0349634)
Supplement: S5 Appendix — (PDF) [file pone.0349634.s005.pdf]

Frequency sweep - 1 lsectin r1 frequency

Storage modulus			Loss modulus			Tan(delta)	Angular frequ	Oscillation tor	Step time	Temperature	Raw phase	Oscillation dis	Complex viscosity
MPa			MPa				rad/s	µN.m	s	°C	°	rad	Pa.s
0,00212164	2,12164	2121,64	0,00282388	2,82388	2823,88	1,33099	0,0628319	498,799	103,112	25	53,0808	0,0209643	56215
0,00181414	1,81414	1814,14	0,00264698	2,64698	2646,98	1,45908	0,0995816	588,307	169,789	24,98	55,5742	0,0272159	32224,7
0,00223697	2,23697	2236,97	0,00301142	3,01142	3011,42	1,3462	0,157827	610,614	213,316	25	53,3939	0,0241641	23768,9
0,00232286	2,32286	2322,86	0,00304869	3,04869	3048,69	1,31247	0,250138	657,724	242,047	24,99	52,697	0,0254761	15322,7
0,0025003	2,5003	2500,3	0,00318838	3,18838	3188,38	1,2752	0,396441	687,941	261,542	25	51,9021	0,0252073	10220,5
0,00263407	2,63407	2634,07	0,00326115	3,26115	3261,15	1,23806	0,628319	716,686	275,032	25	51,0863	0,0253855	6671,88
0,00275289	2,75289	2752,89	0,00334128	3,34128	3341,28	1,21373	0,995816	739,497	284,92	25	50,5517	0,0253713	4347,46
0,00282733	2,82733	2827,33	0,00341937	3,41937	3419,37	1,2094	1,57827	761,451	292,387	25	50,5064	0,0255115	2811,22
0,00284189	2,84189	2841,89	0,00349438	3,49438	3494,38	1,2296	2,50139	777,988	298,589	24,99	51,1117	0,0257268	1800,64
0,00285373	2,85373	2853,73	0,00362725	3,62725	3627,25	1,27106	3,96441	794,914	305,384	24,99	52,3876	0,0257724	1164,17
0,0028221	2,8221	2822,1	0,0037566	3,7566	3756,6	1,33113	6,28319	809,701	311,914	25	54,5625	0,0260704	747,796
0,00279303	2,79303	2793,03	0,00392583	3,92583	3925,83	1,40558	9,95809	815,182	318,678	25,01	58,3572	0,0262433	483,828
0,00269175	2,69175	2691,75	0,00412972	4,12972	4129,72	1,53422	15,7828	804,324	325,38	25,01	67,0049	0,0266163	312,335
0,00274968	2,74968	2749,68	0,00447525	4,47525	4475,25	1,62755	25,0137	801,254	332,036	25	85,0152	0,026479	209,984
0,00305172	3,05172	3051,72	0,00450271	4,50271	4502,71	1,47547	39,6438	976,849	338,566	25	122,558	0,0271458	137,207
0,00334927	3,34927	3349,27	0,00461327	4,61327	4613,27	1,3774	62,8319	2270,11	345,376	25	158,208	0,0271206	90,732
0,00385057	3,85057	3850,57	0,0050054	5,0054	5005,4	1,29991	99,5816	5832,02	351,891	25	171,513	0,025527	63,4167
0,0045998	4,5998	4599,8	0,00541497	5,41497	5414,97	1,17722	157,827	14418	358,545	25,01	176,531	0,0239153	45,0171
0,00645087	6,45087	6450,87	0,00549213	5,49213	5492,13	0,851378	250,14	36468,5	365,216	25	178,629	0,0235826	33,8696
0,0135176	13,5176	13517,6	0,00427461	4,27461	4274,61	0,316226	396,441	92169,2	371,996	25	179,577	0,0236244	35,7616
0,0488201	48,8201	48820,1	-0,00912326	-9,12326	-9123,26	-0,186875	628,319	193141	378,603	25	180,363	0,0199117	79,0448

Frequency sweep - 1		lsectin r2 frequency											
Storage modulus			Loss modulus			Tan(delta)	Angular freque	Oscillation tor	Step time	Temperature	Raw phase	Oscillation dis	Complex viscosity
MPa			MPa				rad/s	µN.m	s	°C	°	rad	Pa.s
0,0015776	1,5776	1577,6	0,00221901	2,21901	2219,01	1,40657	0,0628319	414,159	103,208	25,01	54,5887	0,0248159	43332,3
0,0014474	1,4474	1447,4	0,00221473	2,21473	2214,73	1,53015	0,0995816	473,744	170,04	25	56,8341	0,0292113	26568,7
0,00163733	1,63733	1637,33	0,00251862	2,51862	2518,62	1,53825	0,157827	502,115	213,487	24,99	56,9733	0,0272684	19033,8
0,00169678	1,69678	1696,78	0,00259921	2,59921	2599,21	1,53185	0,250138	537,828	242,215	24,99	56,8664	0,0282685	12409,2
0,00181852	1,81852	1818,52	0,0027713	2,7713	2771,3	1,52393	0,396441	568,893	261,632	25,01	56,7356	0,0280018	8361,08
0,00192395	1,92395	1923,95	0,00289643	2,89643	2896,43	1,50546	0,628319	600,766	275,223	25	56,4274	0,0281938	5534,12
0,00204416	2,04416	2044,16	0,00303437	3,03437	3034,37	1,48441	0,995816	631,54	285,018	24,99	56,0855	0,0281775	3674,06
0,00217552	2,17552	2175,52	0,00317504	3,17504	3175,04	1,45944	1,57827	663,85	292,619	25	55,7071	0,0281807	2438,65
0,00229954	2,29954	2299,54	0,00331678	3,31678	3316,78	1,44236	2,50139	696,39	298,883	24,98	55,5686	0,0282522	1613,48
0,00241323	2,41323	2413,23	0,00347508	3,47508	3475,08	1,44001	3,96441	729,12	305,652	25	55,952	0,0283614	1067,2
0,00253575	2,53575	2535,75	0,00365515	3,65515	3655,15	1,44145	6,28319	759,94	312,26	25,01	57,0157	0,0284518	708,018
0,00267408	2,67408	2674,08	0,00384949	3,84949	3849,49	1,43956	9,95809	782,821	319,117	25,01	59,5474	0,0285996	470,687
0,00281292	2,81292	2812,92	0,00402094	4,02094	4020,94	1,42945	15,7828	778,631	325,944	25	66,1134	0,0288859	310,92
0,00311255	3,11255	3112,55	0,00421067	4,21067	4210,67	1,35281	25,0137	751,504	332,648	25,01	82,965	0,0288979	209,333
0,00337284	3,37284	3372,84	0,00424365	4,24365	4243,65	1,25818	39,6438	957,61	339,303	25,01	126,503	0,0295924	136,737
0,00371227	3,71227	3712,27	0,00445208	4,45208	4452,08	1,19929	62,8319	2415,98	346,083	25	160,61	0,029393	92,2577
0,00430276	4,30276	4302,76	0,00484148	4,84148	4841,48	1,1252	99,5816	6326,88	352,754	25	172,504	0,0278126	65,0438
0,00524804	5,24804	5248,04	0,00524836	5,24836	5248,36	1,00006	157,827	15787,4	359,271	25	176,934	0,0262476	47,0266
0,00791786	7,91786	7917,86	0,00522445	5,22445	5224,45	0,659831	250,14	40048,2	365,972	24,99	178,81	0,0259834	37,9235
0,0169946	16,9946	16994,6	0,00280704	2,80704	2807,04	0,165173	396,441	101181	372,752	25,01	179,747	0,026021	43,4486
0,0532095	53,2095	53209,5	-0,00942896	-9,42896	-9428,96	-0,177205	628,319	191416	379,281	25	180,341	0,0197274	86,0049

Frequency sweep - 1		lsectin r3 frequency											
Storage modulus			Loss modulus			Tan(delta)	Angular freque	Oscillation tor	Step time	Temperature	Raw phase	Oscillation dis	Complex viscosity
MPa			MPa				rad/s	µN.m	s	°C	°	rad	Pa.s
0,00126954	1,26954	1269,54	0,00177127	1,77127	1771,27	1,3952	0,0628319	339,416	103,032	25	54,3686	0,0184689	34683,8
0,00111146	1,11146	1111,46	0,00166611	1,66611	1666,11	1,49903	0,0995816	366,212	169,63	25	56,2927	0,0216821	20112,3
0,0012088	1,2088	1208,8	0,00183218	1,83218	1832,18	1,5157	0,157827	372,339	213,138	25	56,5854	0,0201152	13907,7
0,00120478	1,20478	1204,78	0,00184709	1,84709	1847,09	1,53313	0,250138	386,793	241,976	25	56,8884	0,020799	8816,24
0,00124652	1,24652	1246,52	0,00195214	1,95214	1952,14	1,56608	0,396441	401,014	261,302	25,01	57,4491	0,0205327	5842,4
0,00121691	1,21691	1216,91	0,00197954	1,97954	1979,54	1,62669	0,628319	412,751	274,768	25	58,443	0,0210689	3698,24
0,00120844	1,20844	1208,44	0,00207132	2,07132	2071,32	1,71405	0,995816	423,554	284,672	24,99	59,8008	0,0209573	2408,13
0,00117891	1,17891	1178,91	0,00213783	2,13783	2137,83	1,8134	1,57827	436,06	292,076	25	61,2788	0,0212116	1546,84
0,00114712	1,14712	1147,12	0,00223125	2,23125	2231,25	1,94509	2,50139	449,115	298,106	24,98	63,1746	0,0213	1002,98
0,00112031	1,12031	1120,31	0,00237454	2,37454	2374,54	2,11953	3,96441	468,273	304,995	25	65,6822	0,0213099	662,281
0,00105725	1,05725	1057,25	0,00251005	2,51005	2510,05	2,37413	6,28319	489,142	311,681	24,98	69,5013	0,0216454	433,478
0,00104534	1,04534	1045,34	0,0027815	2,7815	2781,5	2,66084	9,95809	519,273	318,445	25	74,9721	0,0213802	298,395
0,00097703	0,97703	977,03	0,0029967	2,9967	2996,7	3,06715	15,7828	554,051	325,167	24,99	85,6709	0,0218615	199,708
0,0011526	1,1526	1152,6	0,00342164	3,42164	3421,64	2,96862	25,0137	624,56	331,774	24,99	102,072	0,0211659	144,343
0,00154075	1,54075	1540,75	0,00342675	3,42675	3426,75	2,22409	39,6438	872,549	338,323	25	132,976	0,0220913	94,7739
0,00193016	1,93016	1930,16	0,00364867	3,64867	3648,67	1,89034	62,8319	1930,37	345,119	25	159,882	0,0215793	65,6952
0,0024862	2,4862	2486,2	0,0041727	4,1727	4172,7	1,67834	99,5816	4721,41	351,664	25	171,336	0,0202136	48,7764
0,00327858	3,27858	3278,58	0,00471941	4,71941	4719,41	1,43947	157,827	11608,3	358,288	25	176,237	0,0191444	36,4099
0,00524115	5,24115	5241,15	0,0050264	5,0264	5026,4	0,959027	250,14	29214,7	364,989	25,01	178,429	0,0189012	29,0311
0,013076	13,076	13076	0,00374028	3,74028	3740,28	0,286042	396,441	73657,6	371,711	25	179,535	0,0189748	34,3063
0,0342275	34,2275	34227,5	-0,00850233	-8,50233	-8502,33	-0,248407	628,319	184831	378,397	25	180,422	0,0189781	56,1303

Amplitude sweep - 1		sample lsectin r1 amplitud									
Storage modulus		Loss modulus		Tan(delta)	Angular frequ	Oscillation tor	Step time	Temperature	Raw phase	Oscillation dis	Oscillation strain
MPa		MPa			rad/s	µN.m	s	°C	°	rad	%
0,0400147	40,0147	0,00309084	3,09084	0,0772427	6,28319	6,5924	6,12335	25	4,43096	2,2903E-05	0,0104934
0,0385527	38,5527	0,00367304	3,67304	0,0952732	6,28319	9,76674	12,9345	25	5,46038	3,5167E-05	0,0161128
0,0385946	38,5946	0,00361334	3,61334	0,0936229	6,28319	15,2228	19,4952	25	5,36634	5,4762E-05	0,0250906
0,0376457	37,6457	0,00406225	4,06225	0,107907	6,28319	23,8628	26,2124	24,99	6,17978	8,7887E-05	0,0402684
0,0373449	37,3449	0,0034851	3,4851	0,0933221	6,28319	37,2542	32,9297	24,99	5,34989	0,00013852	0,0634674
0,0376088	37,6088	0,00353253	3,53253	0,0939282	6,28319	58,8761	39,5688	25	5,38428	0,00021736	0,0995909
0,0372393	37,2393	0,00366968	3,66968	0,0985434	6,28319	93,1995	46,2079	25,01	5,64739	0,00034735	0,15915
0,0368426	36,8426	0,00375809	3,75809	0,102004	6,28319	146,184	52,8469	25	5,84461	0,00055051	0,252238
0,0360432	36,0432	0,00403171	4,03171	0,111858	6,28319	228,119	59,5172	24,99	6,40529	0,00087728	0,401957
0,0347049	34,7049	0,00461564	4,61564	0,132997	6,28319	351,465	66,1875	25,01	7,6039	0,00140034	0,641623
0,0325457	32,5457	0,00572073	5,72073	0,175775	6,28319	531,484	72,6235	25	10,0089	0,00224395	1,0282
0,0287806	28,7806	0,0078115	7,8115	0,271416	6,28319	777,439	79,1062	25	15,2522	0,0036384	1,66715
0,0221038	22,1038	0,0109414	10,9414	0,495	6,28319	1068,82	85,858	25	26,4751	0,00605125	2,77288
0,0136073	13,6073	0,0119423	11,9423	0,877641	6,28319	1326,64	92,485	25	41,5589	0,0102385	4,69201
0,00768175	7,68175	0,0102472	10,2472	1,33396	6,28319	1509,32	99,0619	24,99	53,6407	0,0164784	7,55175
0,00412735	4,12735	0,0077978	7,7978	1,8893	6,28319	1650,08	105,654	25,01	62,9099	0,0261701	11,9939
0,00220821	2,20821	0,00562896	5,62896	2,54911	6,28319	1772,98	112,186	24,99	69,8168	0,0410666	18,8209
0,00116459	1,16459	0,00393508	3,93508	3,37893	6,28319	1892,53	119,014	25,01	75,3944	0,0646474	29,6283
0,00057746	0,577462	0,00266582	2,66582	4,61645	6,28319	1994,76	125,652	25	80,665	0,102563	47,0058
0,00024812	0,24812	0,00173524	1,73524	6,99354	6,28319	2045,07	132,26	25,01	86,4108	0,163388	74,8821
8,4747E-05	0,0847471	0,0011121	1,1121	13,1226	6,28319	2072,66	138,931	25,01	92,8077	0,258571	118,506

Amplitude sweep - 1		sample lsectin r2 amplitud									
Storage modulus		Loss modulus		Tan(delta)	Angular frequ	Oscillation tor	Step time	Temperature	Raw phase	Oscillation dis	Oscillation strain
MPa		MPa			rad/s	µN.m	s	°C	°	rad	%
0,0504978	50,4978	0,00500642	5,00642	0,0991413	6,28319	7,74338	6,04546	25,01	5,67663	2,2861E-05	0,00974298
0,0528196	52,8196	0,00384457	3,84457	0,0727869	6,28319	12,8606	12,4658	25,01	4,17331	3,6377E-05	0,0155034
0,0527836	52,7836	0,00446075	4,46075	0,0845101	6,28319	20,827	19,136	24,99	4,84251	5,8897E-05	0,0251008
0,0531108	53,1108	0,00419979	4,19979	0,0790761	6,28319	33,1103	25,8377	25	4,53239	9,3096E-05	0,0396758
0,0535637	53,5637	0,0040248	4,0248	0,0751406	6,28319	52,8056	32,5548	25,01	4,30757	0,00014726	0,0627591
0,0535898	53,5898	0,00446064	4,46064	0,0832366	6,28319	84,111	39,2563	25	4,76965	0,0002343	0,0998524
0,0534757	53,4757	0,00469309	4,69309	0,0877612	6,28319	133,292	45,9891	25	5,02766	0,00037194	0,158515
0,0526673	52,6673	0,00549401	5,49401	0,104315	6,28319	209,67	52,503	25	5,96998	0,00059314	0,252784
0,0504919	50,4919	0,00712191	7,12191	0,14105	6,28319	323,817	59,2673	25	8,04938	0,00095135	0,405457
0,0468112	46,8112	0,00912159	9,12159	0,194859	6,28319	487,149	65,8438	25,01	11,0573	0,00153045	0,652289
0,0412862	41,2862	0,0113497	11,3497	0,274903	6,28319	707,139	72,4204	25	15,4199	0,00247512	1,05494
0,0339708	33,9708	0,0136916	13,6916	0,403041	6,28319	983,488	79,0751	24,99	22,0339	0,0040258	1,71596
0,0233376	23,3376	0,0164643	16,4643	0,705482	6,28319	1283,95	85,6673	24,99	35,3697	0,00674272	2,87423
0,0128937	12,8937	0,0153297	15,3297	1,18893	6,28319	1509,82	92,3842	24,99	50,2564	0,0113102	4,82155
0,0072731	7,2731	0,0120905	12,0905	1,66236	6,28319	1659,58	99,1016	25	59,4899	0,0176602	7,52891
0,00400846	4,00846	0,0088516	8,8516	2,20823	6,28319	1805,37	105,725	24,99	66,4425	0,0279191	11,9027
0,00221011	2,21011	0,0062695	6,2695	2,83674	6,28319	1940,45	112,458	25	71,8053	0,0439066	18,7189
0,00118832	1,18832	0,00430628	4,30628	3,62384	6,28319	2058,94	119,082	25,01	76,4389	0,069406	29,59
0,00062044	0,620435	0,00289845	2,89845	4,67164	6,28319	2158,42	125,892	25	80,775	0,109761	46,7949
0,00030075	0,300753	0,00189246	1,89246	6,2924	6,28319	2216,53	132,547	25	85,4373	0,174346	74,3277
0,00013201	0,132008	0,00121323	1,21323	9,19056	6,28319	2246,67	139,061	25,01	90,8372	0,276489	117,877

Amplitude sweep - 1		sample lsectin r3 amplitud									
Storage modulus		Loss modulus		Tan(delta)	Angular frequ	Oscillation tor	Step time	Temperature	Raw phase	Oscillation dis	Oscillation strain
MPa		MPa			rad/s	µN.m	s	°C	°	rad	%
0,0109	10,9	0,00132942	1,32942	0,121966	6,28319	1,66863	5,90526	25	7,02405	1,7026E-05	0,00977231
0,011535	11,535	0,00111101	1,11101	0,0963169	6,28319	2,7876	12,3921	24,99	5,55421	2,6938E-05	0,0154617
0,0112876	11,2876	0,00116051	1,16051	0,102813	6,28319	4,47384	18,9375	25,01	5,92752	4,4162E-05	0,0253473
0,011511	11,511	0,0011525	1,1525	0,100121	6,28319	7,09949	25,5346	25	5,77226	6,8725E-05	0,0394459
0,0114011	11,4011	0,00133776	1,33776	0,117336	6,28319	11,3451	32,0643	25	6,75685	0,00011068	0,0635288
0,0114702	11,4702	0,00131601	1,31601	0,114733	6,28319	17,9141	38,5963	24,99	6,60793	0,00017376	0,0997335
0,0112457	11,2457	0,00144305	1,44305	0,12832	6,28319	28,2075	45,3965	25	7,38369	0,00027865	0,15994
0,0109799	10,9799	0,0015485	1,5485	0,14103	6,28319	43,7564	51,9759	25,01	8,10772	0,00044206	0,253734
0,0105548	10,5548	0,00174006	1,74006	0,16486	6,28319	67,2934	58,7915	24,99	9,45853	0,00070495	0,40463
0,00965892	9,65892	0,0020576	2,0576	0,213026	6,28319	100,347	65,3054	25	12,1605	0,00113954	0,654075
0,00746715	7,46715	0,00287098	2,87098	0,384482	6,28319	136,539	71,804	25	21,3189	0,00191779	1,10081
0,00554206	5,54206	0,00301613	3,01613	0,544226	6,28319	172,593	78,555	25,01	29,0449	0,0030814	1,76872
0,0030301	3,0301	0,00313672	3,13672	1,03519	6,28319	203,133	85,1942	24,99	47,0584	0,00525778	3,01813
0,00158674	1,58674	0,00245252	2,45252	1,54564	6,28319	215,839	91,7552	25	58,966	0,00836347	4,80081
0,00084019	0,840188	0,00177856	1,77856	2,11686	6,28319	226,819	98,316	25	67,7117	0,0130868	7,51225
0,00043884	0,438841	0,00123901	1,23901	2,82337	6,28319	238,441	104,972	25,01	75,1874	0,0206335	11,8443
0,00021998	0,219978	0,00084372	0,843723	3,83549	6,28319	249,773	111,659	24,99	82,6535	0,0325618	18,6916
0,00010013	0,100131	0,00056244	0,562443	5,61708	6,28319	261,424	118,173	25	91,1277	0,0515382	29,5844
3,9201E-05	0,0392006	0,00037508	0,375083	9,5683	6,28319	276,941	124,625	25	100,867	0,0804165	46,1617
2,0592E-05	0,0205922	0,00026088	0,260882	12,669	6,28319	297,973	131,405	25	109,154	0,119659	68,6879
7,8312E-06	0,00783116	0,00017283	0,172829	22,0693	6,28319	337,718	138,122	25	120,884	0,185978	106,761
